# MicroRNA-18a promotes cancer progression through SMG1 suppression and mTOR pathway activation in nasopharyngeal carcinoma

**DOI:** 10.1038/s41419-019-2060-9

**Published:** 2019-10-28

**Authors:** ShiJuan Mai, RuoWen Xiao, Lu Shi, XiaoMin Zhou, Te Yang, MeiYin Zhang, NuoQing Weng, XinGe Zhao, RuiQi Wang, Ji Liu, Rui Sun, HaiDe Qin, HuiYun Wang

**Affiliations:** 1 State Key Laboratory of Oncology in South China, Collaborative Innovation Center for Cancer Medicine, Guangdong Key Laboratory of Nasopharyngeal Carcinoma Diagnosis and Therapy, Sun Yat-sen University Cancer Center, Guangzhou, 510060 China; 2 Department of thoracic oncology, the cancer center of the fifth affiliated hospital of Sun Yat-sen University, Zhuhai, 519000 China; 3 ZhouKou Hospital of Traditional Chinese Medicine, Zhoukou, 466000 China; 4 Guangdong Provincial Key Laboratory of Malignant Tumor Epigenetics and Gene Regulation, Sun Yat-sen Memorial Hospital, Sun Yat-sen University, Guangzhou, 510120 China; 5 Department of Nasopharyngeal Carcinoma, Sun Yat-sen University Cancer Center, Guangzhou, 510060 China

**Keywords:** Head and neck cancer, miRNAs

## Abstract

miR-18a has been reported to be upregulated in nasopharyngeal carcinoma (NPC) tissues by microarray assays. However, the roles and the underlying mechanisms of miR-18a in NPC remain poorly understood. Here we demonstrated by real-time RT-PCR that miR-18a expression is upregulated in NPC tissues, and positively correlated with tumor size and TNM stage. Moreover, miR-18a expression could be upregulated by NF-κB activation or Epstein-Barr virus encoded latent membrane protein 1 expression. The ectopic expression of miR-18a promoted NPC cell proliferation, migration and invasion, while the repression of miR-18a had opposite effects. Candidate genes under regulation by miR-18a were screened out through a whole-genome microarray assay, further identified by a reporter assay and verified in clinical samples. SMG1, a member of the phosphoinositide 3-kinase-related kinases family and an mTOR antagonist, was identified as functional target of miR-18a. Our results confirmed that miR-18a exerts its oncogenic role through suppression of SMG1 and activation of mTOR pathway in NPC cells. Importantly, in vivo xenograft tumor growth in nude mice was effectively inhibited by intratumor injection of miR-18a antagomir. Our data support an oncogenic role of miR-18a through a novel miR-18a/SMG1/mTOR axis and suggest that the antitumor effects of antagomir-18a may make it suitable for NPC therapy.

## Introduction

Nasopharyngeal carcinoma (NPC) is particularly common in Southern China and southeastern Asia, where the age-standardized incidence peaks at 30 per 100,000 person-years^1,2^. The multifactorial etiologies of NPC include genetic predisposition, Epstein-Barr virus (EBV) infection, and environmental factors^3^. Due to a highly invasive and metastatic character, 75-85% of NPC patients have metastatic nodes develop during the course of disease^4^. Although NPC is sensitive to radiotherapy and precise radiotherapy technologies and combined chemotherapy have improved the local control of NPC, the outcome of NPC in advanced stages is still poor, with the 5-year survival rate ranging from 41 to 63%^5,6^. Thus, there is an urgent need to explore the key factors leading to the progression of NPC and to develop additional targeted therapies for NPC.

MicroRNAs are known to play a pivotal role in tumorigenesis and cancer progression in humans. In particular, miR-17-92, a polycistron encoding six mature miRNAs, namely, miR-17, miR-18a, miR-19a, miR-20a, miR-19b, and miR-92a, is among the most potent oncogenic miRNAs^7^. Despite the overexpression of the miR-17-92 cluster in multiple cancer types, other studies suggest that the miR-17-92 cluster undergoes a loss of heterozygosity and acts as a tumor suppressor in several different cancers^8^. Previous studies using miRNA microarray assays on NPC biopsies revealed that miR-18a, derived from the miR-17-92 cluster, was significantly upregulated in NPC tissues and might play important roles in the development of NPC^9–11^. However, the role of miR-18a in NPC and the underlying molecular mechanism by which miR-18a promotes NPC development remain incompletely understood; only one recent study has reported that miR-18a could promote NPC cell proliferation by targeting the miRNA-processing enzyme DICER1^12^.

Here, we demonstrate that miR-18a (also named miR-18a-5p) expression is upregulated in NPC tissues and is positively correlated with tumor size and TNM stage of NPC by using a quantitative real-time RT-PCR (qRT-PCR) assay on NPC biopsies. The ectopic expression of miR-18a in NPC cells promotes cell survival, epithelial mesenchymal transition (EMT) and invasion. Furthermore, we provide evidence that miR-18a promotes the activation of the mTOR pathway by directly targeting SMG1 gene expression. In addition, we have shown that miR-18a expression can be induced by EBV encoded latent membrane protein 1 (LMP1) or NF-κB activation. Thus, the results reveal a novel mechanism by which miR-18a-mediated signaling facilitates the progression of NPC cells.

## Materials and methods

### Cell lines, antibodies, and reagents

The human NPC cell lines CNE1, CNE2, S-18, S-26, 5-8F, 6-10B, SUNE2 and C666-1 cells were preserved in our cancer center and routinely maintained in RPMI-1640 containing 100 units/ml penicillin, 100 µg/ml streptomycin, and 10% FBS (Invitrogen, CA, USA) at 37 °C in a 5% CO_2_ humidified atmosphere. HK1 cells were cultured in RPMI1640 supplemented with 5% FBS. NP69, an immortalized human nasopharyngeal epithelial cell line, was cultured in defined keratinocyte serum-free medium supplemented with epidermal growth factor (Invitrogen, CA, USA). C666-1 is the only in vitro native EBV-infected NPC cell line. Other NPC cell lines have lost EBV in long-term cultures thus are EBV-negative. All of the normal nasopharyngeal epithelial cell lines and NPC cell lines were generously provided by Professor MuSheng Zeng (Sun Yat-sen University Cancer Center) with authenticated using short tandem repeat profiling, tested for *Mycoplasma* contamination.

Mouse monoclonal antibodies against human E-cadherin, N-cadherin and Vimentin were purchased from BD Biosciences (BD Transduction Laboratories, Lexington, UK). Rabbit polyclonal antibody against Snail was purchased from Proteintech (Wuhan, China). Rabbit monoclonal antibodies against human phosphop70S6K (Thr389), p70S6K, phospho-4E-BP1, and 4E-BP1 were obtained from Cell Signaling Technology (Danvers, MA, USA). Mouse monoclonal antibodies against EBV LMP1 and SMG1 were purchased from Abcam (Cambridge, UK).

To induce or inhibit NF-κB activity, NPC cells were treated with 10 ng/ml TNF-α (Sigma, St Louis, MO, USA) or 2.5 μM BAY 11-7082 (Selleck Chemicals, Houston, TX, USA), respectively, before luciferase analysis or evaluation of miR-18a expression by qRT-PCR. For rapamycin treatment, cells were pretreated with 20 ng/ml of rapamycin (Selleck Chemicals, Houston, TX, USA) for 30 min before the following experiments.

### Clinical samples

Twenty-one cases of fresh NPC tissues and 14 non-cancerous nasopharyngitis (NP) tissues were used for qRT-PCR detection of miR-18a. Twelve paired fresh NPC tissues and adjacent noncancerous nasopharyngeal mucosal tissues were used for qRT-PCR detection of SMG1 and miR-18a. All fresh samples were collected at the time of diagnosis and preserved in liquid nitrogen until further use. Formalin-fixed paraffin-embedded tissues of 67 primary NPC tissues were obtained from the archives of the Department of Pathology in the Cancer Center, Sun Yat-sen University, between January 2007 and December 2008. All patients were histologically and clinically diagnosed as NPC, assessed according to the TNM staging of International Union against Cancer. None of the patients received radiotherapy or chemoradiotheray before biopsy sampling. This study was approved by the Research Ethics Committee of Sun Yat-sen University Cancer Center, and written informed consent was obtained from each participant.

### Vectors and transfection

Lentivirus overexpressing miR-18a was purchased from GenePharma (Shanghai, China). NPC cells were infected with recombinant lentivirus transducing units plus 8 mg/ml Polybrene (Sigma, St Louis, Missouri, USA). Stable cell lines were selected using 4 μg/mL puromycin. Transient transfection was performed using Lipofectamine 2000 reagent (Invitrogen, CA, USA) in OPTI-MEM media. miR-18a mimic, miR-18a inhibitor, siRNA against LMP1 or SMG1 and their negative controls were obtained from RiboBio (Guangzhou, China). pcDNA3.1(+)-LMP1 plasmid was kindly provided by Professor BiJun Huang (Sun Yat-sen University Cancer Center).

### RNA extraction and qRT-PCR

Total RNA was extracted from cell lines and fresh tissues with TRIzol reagent (Invitrogen, CA, USA) or paraffin-embedded tissues with phenol chloroform according to the manufacturer’s instructions. cDNA was synthesized with the PrimeScript RT reagent Kit (Promega, Madison, WI, USA). Quantitative real-time PCR analysis was carried out using TaqMan Reverse Transcription Kits and the TaqMan Assays (Life Technologies, Darmstadt, Germany). U6 and GAPDH were used as the internal controls for the quantification of miR-18a and SMG1, respectively. Quantitative RT-PCR was carried out on the Roche LightCycler® 96 real-time PCR platform, and gene expression was quantified using the 2^−ΔΔCT^ method.

### Western blot

Total protein was extracted from cultured cells using RIPA buffer containing PMSF and quantified using a BCA protein assay kit (Beyotime, Haimen, China). Protein lysates were subjected to SDS-PAGE and transferred onto polyvinylidenedifluoride membranes (Millipore, Billerica, MA), followed by incubation first with a primary antibody and then with a secondary antibody. The signals were detected with the KeyGEN Enhanced ECL detection kit according to the manufacturer′s instructions (KeyGEN, NanJing, China).

### Microarray assay

miR-18a knockdown 5-8F cells and miR-18a over-expressing 6-10B cells and their corresponding control cells were selected for microarray analysis. Total RNA was isolated from cell lines using TRIzol reagent (Invitrogen, CA, USA) according to the manufacturer’s instructions. RNA quantity and quality were measured by NanoDrop ND-1000. RNA integrity was assessed by standard denaturing agarose gel electrophoresis. HumanGene Expression 4 × 44k v2 Microarray Kit (Agilent Technologies) were used to monitor changes in gene expression. The microarray analysis was performed by Kangchen Bio-tech Inc (Shanghai, China). Sample labeling and array hybridization were performed according to the Agilent One-Color Microarray-Based Gene Expression Analysis protocol (Agilent Technology). Agilent Feature Extraction software (version 11.0.1.1) was used to analyze acquired array images. Quantile normalization and subsequent data processing were performed with using the GeneSpring GX v12.1 software package (Agilent Technologies). Our microarray studies utilize no replication due to practical considerations of cost. Therefore, the threshold of two-fold change was defined to identify differentially expressed genes and qRT-PCR was performed to validate and quantify the differentially expressed genes related with PI3K/AKT/mTOR or EMT network to verify the reliability of the microarray results. The qRT-PCR results were generally consistent with the microarray data.

### Immunohistochemistry (IHC)

IHC was performed using a standard streptavidin-biotin-peroxidase complex method. In brief, slides with paraffin-embedded sections were deparaffinized and rehydrated. Endogenous peroxidase activity was blocked with 0.3% hydrogen peroxide for 15 min. For antigen retrieval, the slides were microwave-treated and boiled in a 10 mM citrate buffer (pH 6.0) for 10 min. The slides were incubated overnight at 4 °C with primary antibody. The staining results were evaluated independently by two pathologists.

### Cell viability assay

Cell viability was detected by the CCK8 Cell Counting Kit (JingXin Biological Technology, Guangzhou, China). Cells were plated and cultured in 96-well plates at a density of 1.0 × 10^3^ cells/well. The detection was performed at the same time every day. The OD value was measured at 450 nm by a microplate reader (SpectraMax® M5 Multimode Microplate Reader; Molecular Devices, LLC, Sunnyvale, CA, USA). All experiments were performed in triplicate.

### Colony formation assay

Cells were placed in six-well plates (300 cells/well) and cultured for 2 weeks. Colonies were fixed with methanol and stained with 0.1% crystal violet in 20% methanol for 15 min. Colonies larger than 0.1 mm in diameter were scored. The experiment was performed in triplicate for each cell line.

### Wound healing and invasion assays

Cell migration was measured by a scratch wound-healing assay. The culture medium was replaced with serum-free DMEM after the generation of a scratch wound, and wound closure was photographed 24 h later. The scratch closures were later analyzed and are represented as percent closure in the figures. For invasion assays, 5 × 10^4^ cells were plated in a Matrigel-coated Transwell chamber (BD Biosciences, Lexington, UK) with an 8 μm pore size. The noninvading cells were removed 16 h later, and the invaded cells on the bottom of the chamber were fixed with 100% methanol and stained with crystal violet. The experiments were performed in triplicate.

### Animal study

Six-week-old male athymic BALB/c nude mice were injected subcutaneously with 4 × 10^6^ control or miR-18a-overexpressing HK1 cells (*n* = 5 per group). Randomization was conducted. Tumor sizes were measured using calipers every 4 days using an unblinded manner, and tumor volumes were calculated (*V* = 0.5 × L × W^2^). Mice were sacrificed 40 days after inoculation and tumors were fixed in formalin, embedded in paraffin and used for hematoxylin-eosin (H&E) staining and IHC assays. For antagomir treatment, 4-week-old male athymic BALB/c nude mice were injected subcutaneously with 6 × 10^6^ 5-8F cells. Antagomirs were synthesized by RiboBio Co. (Guangzhou, China). After 15 days, when the tumors became palpable, the tumor-bearing mice were assigned to two groups (five mice per group) randomly. 20 nmol antagomir-18a for test group or negative control for control group was injected intratumorally in a multisite injection manner every 3 days for 2 weeks. Tumor volumes and weights were calculated as described above. All animal studies were approved by the laboratory animal ethics committee of Sun Yat-sen University Cancer Center.

### Luciferase reporter assay

miR-18a belongs to the miR-17–92 cluster. The potential NF-κB binding sites were predicted in the 2000 bp upstream region of the transcription start site (chr13: 72,977,942-72,979,921) of miR-17–92 by the TFSEARCH (http://www.cbrc.ip/research/db/ TFSEARCH.html) and MOTIF (http://motif.genome.jp/). Next, three miR-17–92 promoters (–1747 to –1583, –1583 to –1328, and –1328 to –790) that spanning the putative NF-κB binding sites at –1698 (TGGAATTTCC), –1442 (TGGGATTTCC) and –827 (CGGAATTTCC) respectively were constructed by PCR using primers reported by Zhou et al.^13^, and transfected into 5-8F cells. The transfected cells were treated with TNF-α or BAY 11-7082 respectively for 24 h followed by measurement of luciferase activity, which was presented as the ratio of the activity of the test construct with the control pGL4-basic vector. All constructs were confirmed by sequencing.

Oligonucleotides containing the wild-type (Wt) or mutant (Mut) putative miR-18a binding sites in the 3′-untranslated regions (3′-UTR) of SMG1 mRNA were separately ligated into the pMIR-REPORT vector (Ambion, Carlsbad, CA). 6-10B cells were transfected with luciferase constructs in the presence of a miR-18a mimic or negative control.

For reporter assay, a total of 4 × 10^4^ cells per well were seeded in 24-well plates in triplicate. Twenty-four hours later, 100 ng of firefly luciferase construct was cotransfected with 10 ng of pRL-TK renilla plasmid into cells using Lipofectamine 2000 reagent. Media were replaced at 6 h, and the luciferase and renilla signals were measured 48 h after transfection using the Dual Luciferase Reporter Assay Kit (Promega, Madison, WI, USA) according to the manufacturer’s protocol. The experiments were performed independently in triplicate.

### Statistical analysis

Sample size was chosen based on the need for statistical power. All experiments were repeated three times or more and data are presented as mean ± SD and Student’s *t*-test (unpaired, two-tailed) was used to evaluate significant differences between groups of experimental data where appropriate. The variance between the groups that are being statistically compared is similar. The Chi-square test was used for correlation analysis between clinicopathological features of patients with NPC and miR-18a expression profiles. The linear correlations between miR-18a and SMG1 mRNA expression in NPC tissues were evaluated with Pearson correlation coefficient analysis. In addition, repeated measure analysis of variance was used to compare the difference of tumor volume between groups of mice treated with antagomirs during the subsequent measurements. The SPSS version 17.0 statistical software package was used for statistical analyses. *P* < 0.05 was regarded as statistically significant (*P < 0.05; **P < 0.01; ***P < 0.001).

## Results

### miR-18a is overexpressed in NPC and is associated with tumor stage

**Fig. 1:**
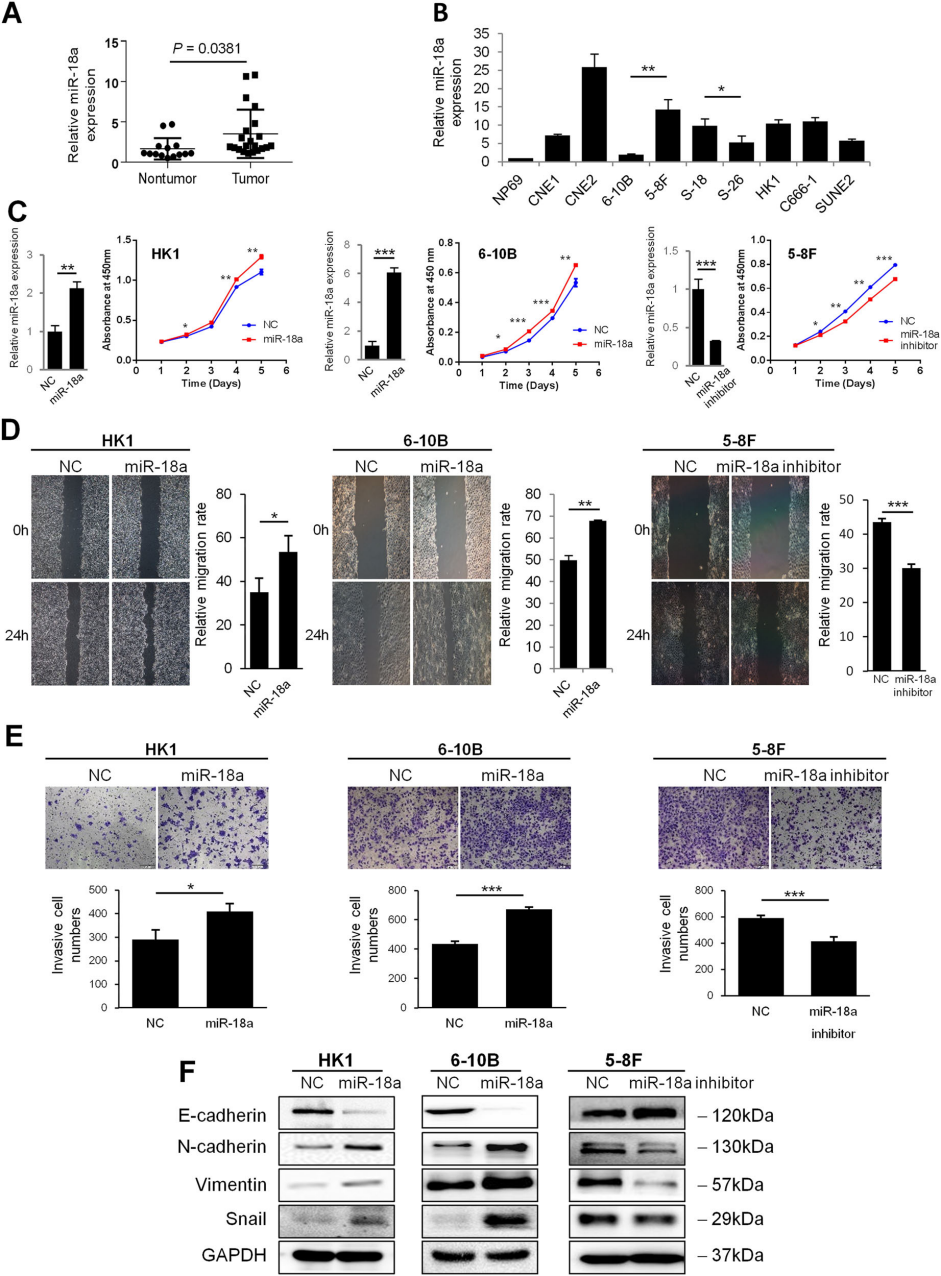
miR-18a is overexpressed in human NPC and promotes cell proliferation, migration and invasion. **a** Expression levels of miR-18a in 21 NPC tissues and 14 noncancerous nasopharyngeal tissues were examined by qRT-PCR. **b** Expression of miR-18a in nine human NPC cell lines relative to the non-neoplastic cell line NP69. HK1 and 6-10B cells stably overexpressing miR-18a and 5-8F cells with transient knockdown of miR-18a were examined for **c** miR-18a expression by qRT-PCR analysis and proliferation by a CCK8 assay and **d** cell migration ability by a scratch wound-healing assay, **e** cell invasion ability by Boyden chamber assays with Matrigel, and **f** expression of EMT markers by western blotting. Data are presented as the mean ± SD. All assays were done in triplicate, and the values represent the mean of three independent experiments. *P < 0.05; **P < 0.01; ***P < 0.001

The expression level of miR-18a was evaluated in 21 snap-frozen NPC tissues and 14 noncancerous nasopharyngitis (NP) tissues by qRT-PCR. The results showed that the miR-18a expression level was significantly upregulated in tumor compared with non-tumor tissues (*P* = 0.0381, Fig. 1a).The miR-18a expression level was further determined in the immortalized non-neoplastic cell line NP69 and a panel of NPC cell lines (CNE1, CNE2, 6-10B, 5-8 F, S-18, S-26, HK1, C666-1, and SUNE2) (Fig. 1b). It was shown that the basal expression level of miR-18a was lowest in NP69 cells. 5-8F (high tumorigenic and metastatic potential) and 6-10B (low tumorigenic and metastatic potential) are paired cell lines derived from the NPC cell line SUNE-1, and S18 (high tumorigenic and metastatic potential) and S26 (low tumorigenic and metastatic potential) were derived from CNE2 cells. The miR-18a expression level was significantly higher in 5-8F and S-18 cells than in their paired subclones 6-10B and S-26 cells.

To determine the correlation of miR-18a level with the clinicpathological features in NPC patients, qRT-PCR was performed on an additional 67 formalin-fixed, paraffin-embedded primary NPC tissues. The expression levels of miR-18a were classified into high (*n* = 34) and low level (*n* = 33) groups according to the median value of miR-18a expression. The clinicpathological parameters of the 67 NPC patients were summarized in Table 1. As shown, high miR-18a expression was positively associated with tumor size (*P* = 0.0021) and clinical stage (*P* = 0.0049). No significant associations were found between miR-18a expression and the other clinicopathological features. These results suggest that miR-18a plays a promoting role in NPC progression.

**Table 1:** Relationship between miR-18a expression and clinicopathologic parameters of 67 NPC

Characteristics	Number of cases	miR-18a expression		*P* value
		Low, *n* (%)	High, *n* (%)	
Age				
≤ 45 years	23	15 (44)	8 (24)	0.0867
>45 years	44	19 (56)	25 (76)	
Gender				
Male	48	26 (76)	22 (67)	0.3734
Female	19	8 (24)	11 (33)	
EBV DNA copy				
≤4000	30	17 (50)	13 (39)	0.3828
>4000	37	17 (50)	20 (61)	
EA-IgA				
<1:10	22	14 (41)	8 (24)	0.1400
≥1:10	45	20 (59)	25 (76)	
VCA-IgA				
<1:80	18	12 (35)	6 (18)	0.1141
≥1:80	49	22 (65)	27 (82)	
Histology, WHO type				
III	66	34 (100)	32 (97)	
II	0	0	0	
I	1	0	1 (3)	
T stage				
T1–T2	31	22 (65)	9 (27)	**0.0021**
T3–T4	36	12 (35)	24 (73)	
N stage				
N0–N1	34	20 (59)	14 (42)	0.1795
N2–N3	33	14 (41)	19 (58)	
M stage				
M0	40	24 (71)	16 (48)	0.0652
M1	27	10 (29)	17 (52)	
TNM stage				
I–II	21	16 (47)	5 (15)	**0.0049**
III–IV	46	18 (53)	28 (85)	

*VCA-IgA*, viral capsid antigen immunoglobulin A; *EA-IgA*, early antigen immunoglobulin A

Significant *P* values were shown in bold

### miR-18a promotes NPC cell growth, migration, and invasion

Stably miR-18a -expressing HK1 and 6-10B cells were constructed using lentiviral transfection and endogenous miR-18a was knocked down in 5-8F cells with an antisense oligonucleotide inhibitor. miR-18a expression was validated by qRT–PCR (Fig. 1c). The CCK8 assay showed that miR-18a overexpression significantly increased the growth rate in both NPC cell lines compared with that in the control cells, whereas miR-18a inhibitor retarded cell growth in 5-8F cells (Fig. 1c).

In wound-healing assays, the motility of HK1-miR-18a and 6-10B-miR-18a was remarkably increased compared with that of the control cells at 24 h post-wounding (Fig. 1d). Next, cell invasion ability was evaluated through a Matrigel-coated Transwell insert. Stable overexpression of miR-18a increased the invasion ability of HK1 and 6-10B cells (Fig. 1e). Conversely, transient transfection of 5-8F cells with a miR-18a inhibitor substantially decreased cell migration and invasion ability (Fig. 1d, e).

To further investigate the molecular mechanism mediating the aggressive effect of miR-18a, we examined the potential regulation of miR-18a on EMT. Western blot analysis revealed that the mesenchymal markers N-cadherin, Vimentin and core EMT transcription factor Snail were significantly upregulated, and the expression of the epithelial marker E-cadherin was decreased, in the miR-18a-expressing HK1 and 6-10B cells. The opposite results were obtained in the 5-8F cells transfected with a miR-18a inhibitor (Fig. 1f).

Taken together, these data showed that miR-18a enhanced malignant phenotypes in NPC cells in vitro.

### Identification of the miR-18a target genes

**Fig. 2:**
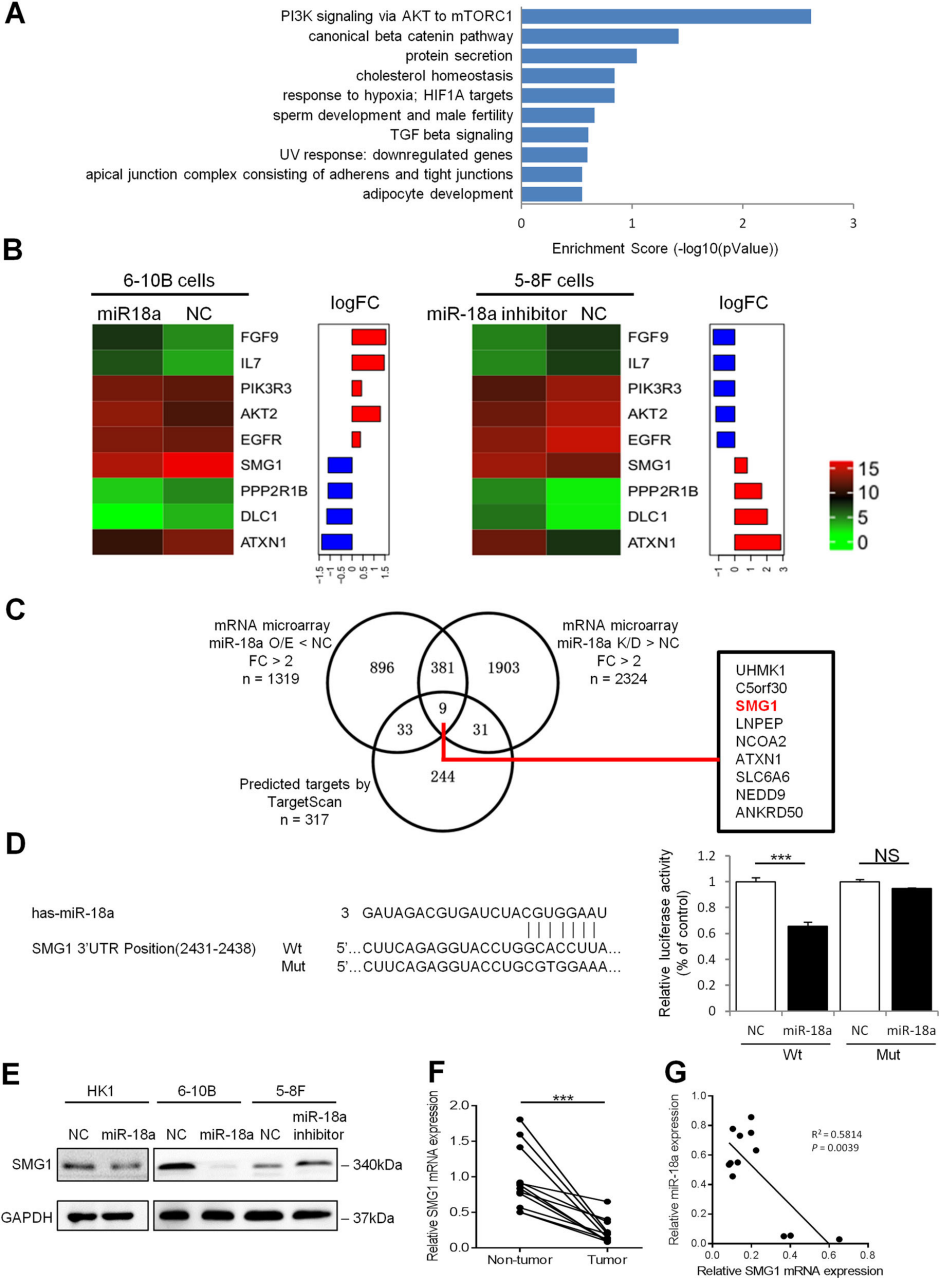
Microarray analysis reveals miR-18a targeted SMG1 gene and PI3K/AKT/mTOR signaling pathway. **a** GSEA reveals miR-18a impacted several pathways involved in PI3K/AKT/mTOR signaling and EMT network, **b** Heat map of representative molecules related with PI3K/AKT/mTOR signaling and EMT network from microarray results, **c** Venn diagram of the candidate miR-18a regulated mRNAs based on the mRNA microarray analysis of miR-18a-overexpressing and knockdown cells and targets predicted by the TargetScan. **d** Left, sequence alignment of miR-18a with binding sites in the SMG1 3′ UTR. Right, the luciferase reporter assay was performed in 6-10B cells transfected with pMIR-REPORT vectors containing either the wild-type or the mutated 3′ UTRs of SMG1 and a miR-18a mimic or negative control. ***P < 0.001. NS: not significant. **e** Western blot assay of SMG1 protein levels in HK1, 6-10B and 5-8F cells and their corresponding control cells 48 h after transfection with a miR-18a mimic or inhibitor. **f** Expression of SMG1 mRNA in 12 paired NPC tissues and adjacent nontumor nasopharyngeal mucosal tissues. ***P < 0.001. **g** Correlation analysis of miR-18a and SMG1 mRNA expression in 12 NPC patients (Pearson correlation, *P* *=* 0.0039)

To search for molecular processes underlying miR-18a-mediated oncogenesis in NPC cells, whole-genome mRNA microarray analysis was performed using the HumanGene Expression 4 × 44k v2 Microarray Kit (Agilent Technologies) in 6-10B cells with stable miR-18a overexpression and in 5-8F cells with transient silencing of miR-18a by siRNA. We identified 2622 differentially expressed genes (fold change > 2), namely, 1319 down- and 1303 upregulated genes, in miR-18a-overexpressing cell and 4159 differentially expressed genes (fold change > 2), namely,1835 down- and 2324 upregulated genes, in miR-18a knockdown cells. Differentially expressed genes (*n* = 390) that simultaneously downregulated in the miR-18a-overexpressing cells and upregulated in the miR-18a-suppressed cells were subjected to Gene Set Enrichment Analysis^14^ (Fig. 2a). We found that the top-scoring canonical pathways include PI3K/AKT/mTOR and EMT related signaling pathway. Negative regulator of the PI3K/AKT/mTOR or EMT network including SMG1, PPP2R1B, DLC1 and ATXN1 were reduced by miR-18a, PI3K/AKT/mTOR pathway components including AKT2 and PIK3R3 or upstream regulator EGFR, FGF9 and IL7 were induced by miR-18a (Fig. 2b). To verify the reliability of the microarray results, qRT-PCR was performed to validate and quantify the differentially expressed genes related with PI3K/AKT/mTOR or EMT network, including five that were upregulated (FGF9, IL7, PIK3R3, AKT2, and EGFR) and four that were downregulated (SMG1, PPP2R1B, DLC1, and ATXN1) by miR-18a. Primers used for qRT-PCR were listed in the Supplementary Table 1. The qRT-PCR results of these gens were generally consistent with the microarray data (Supplementary Fig. 1). To identify putative targets of miR-18a in NPC cells, we integrated the differentially expressed genes from our microarray approaches (*n* = 390) and predicted miR-18a targets by TargetScan (release 7.1) (*n* = 317). Nine genes including UHMK1, C5orf30, SMG1, LNPEP, NCOA2, ATXN1, SLC6A6, NEDD9, and ANKRD50 were selected as the candidate targets of miR-18a (Fig. 2c). SMG1 (nonsense-mediated mRNA decay associated PI3K related kinase) has been reported to be a member of the phosphoinositide 3-kinase-related kinases family and act as an mTOR antagonist. Therefore, we particularly focused on SMG1 in the following experiments. The expression profile of SMG1 in microarray data was summarized in Supplementary Table 2.

### SMG1 is directly targeted by miR-18a and downregulated in NPC

Putative miR-18a target sites in the 3′UTR of SMG1 mRNA were predicted by the TargetScan (Fig. 2d, left). To validate SMG1 as a direct target of miR-18a, reporter plasmids containing the wild-type or target site-mutated 3′UTR fragments of SMG1 were constructed. As expected, the luciferase activities of the reporter containing the wild-type 3′UTR in 6-10B cells were reduced significantly after transfection with miR-18a mimics. No obvious change in luciferase expression was found in cells transfected with reporters containing mutations in the miR-18a binding sites in the SMG1 3′UTR (Fig. 2d, right). In agreement with the bioinformatic predictions and reporter assay, the protein level of SMG1 was downregulated in HK1 and 6-10B cells in response to the miR-18a mimic and upregulated in 5-8F cells transfected with the miR-18a inhibitor (Fig. 2e). These results suggest that SMG1 is a direct functional target of miR-18a in NPC.

To explore the expression level of SMG1 in NPC patients, we compared SMG1 mRNA expression levels in 12 paired fresh NPC tissues and adjacent noncancerous nasopharyngeal mucosal tissues by using qRT-PCR. The results showed that SMG1 was significantly downregulated in tumor tissues compared with non-tumor tissues (*P* < 0.001) (Fig. 2f). Moreover, an inverse correlation between miR-18a and SMG1 mRNA expression was determined in 12 NPC tissue samples by using qRT-PCR analysis (*P* = 0.0039, Fig. 2g).

### miR-18a exerts oncogenic effects through the suppression of SMG1

**Fig. 3:**
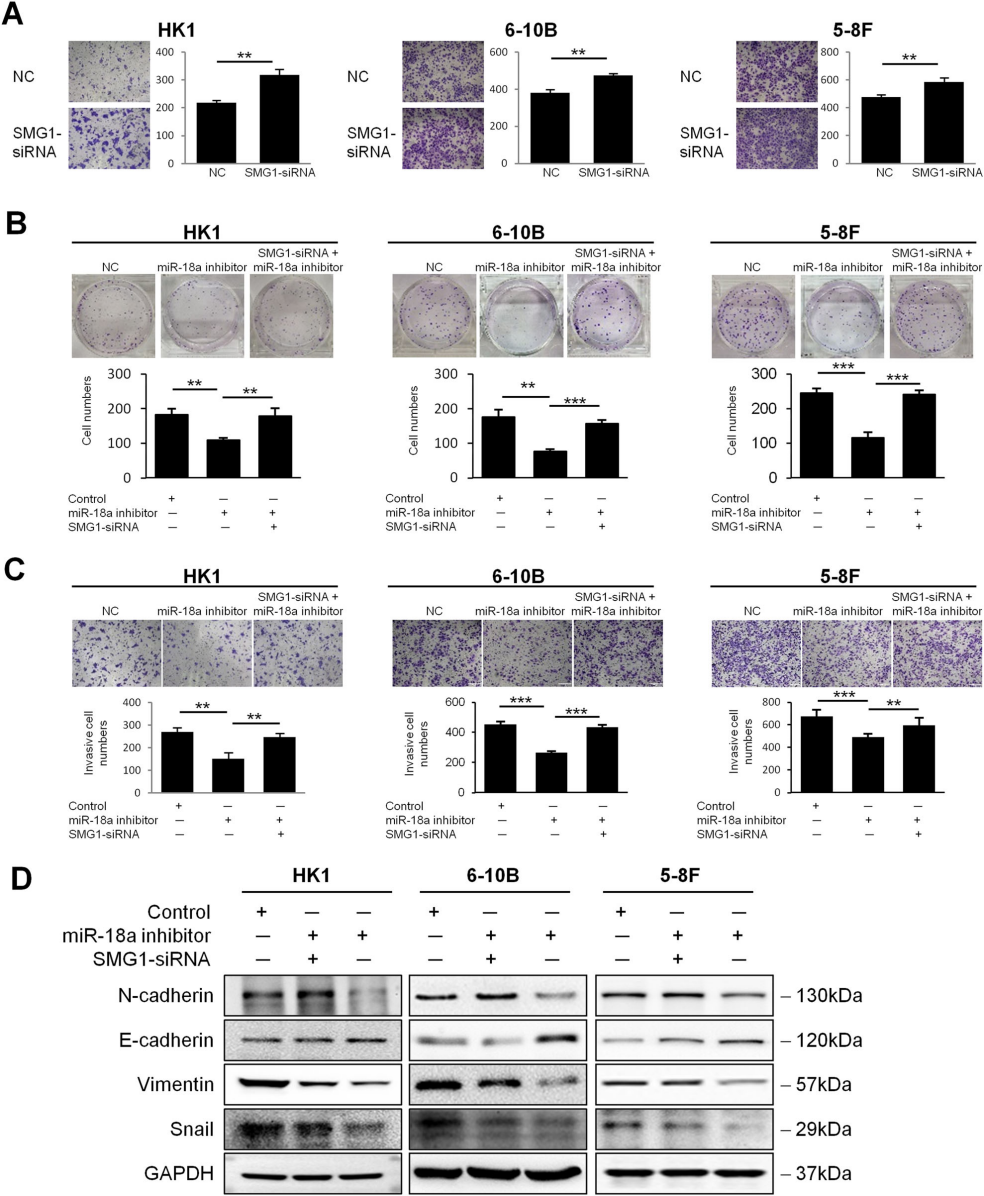
miR-18a exerts oncogenic effects through the SMG1 suppression. **a** Invasion assay with HK1, 6-10B and 5-8 F cells transfected with siRNA against SMG1 or with a negative control. Suppression of SMG1 expression by siRNA restored the reduced colony formation (**b**), invasion ability (**c**), and EMT phenotypes (**d**) in miR-18a inhibitor-treated HK1, 6-10B and 5-8F cells. Data are presented as the mean ± SD. All assays were done in triplicate, and the values represent the mean of three independent experiments. **P < 0.01; ***P < 0.001

As miR-18a has been shown to promote NPC cells migration and invasion, we hypothesized that SMG1, as a predicted target gene of miR-18a, might play an opposite role in NPC. To test this hypothesis, endogenous SMG1 in HK1, 6-10B, and 5-8F cells was silenced by SMG1-specific siRNA oligos. The Matrigel-coated Transwell assay showed that the average number of invading HK1, 6-10B, and 5-8F cells transfected with siRNAs against SMG1 was significantly higher than that of cells treated with control siRNA (Fig. 3a).

Next, we set out to corroborate whether SMG1 suppression mediates the role of miR-18a in cancer cells. For the rescue experiment, it is better to cotransfect miR-18a mimics and SMG1-expressing plasmid into NPC cells. Unfortunately, we failed to construct SMG1 overexpressing plasmid since the coding sequence of SMG1 gene (NM_015092.4) reach 10,986 bp in length. Alternatively, SMG1-targeting siRNA and miR-18a inhibitors were cotransfected into HK1, 6-10B and 5-8F cells, respectively. Whereas miR-18a suppression inhibited the potential for colony formation and invasion, simultaneous treatment with SMG1 siRNA recovered the oncogenic phenotypes (Fig. 3b, c). Furthermore, western blot analysis demonstrated that EMT induction was restored by the introduction of SMG1 siRNAs into HK1, 5-8F and 6-10B cells transfected with the miR-18a inhibitor (Fig. 3d). These results suggest that SMG1 induction mediates the effects of miR-18a inhibition in cancer cell migration and invasion.

### SMG1-dependent mTOR signaling activation is essential for miR-18a-stimulated promotion of cellular motility in NPC cells

**Fig. 4:**
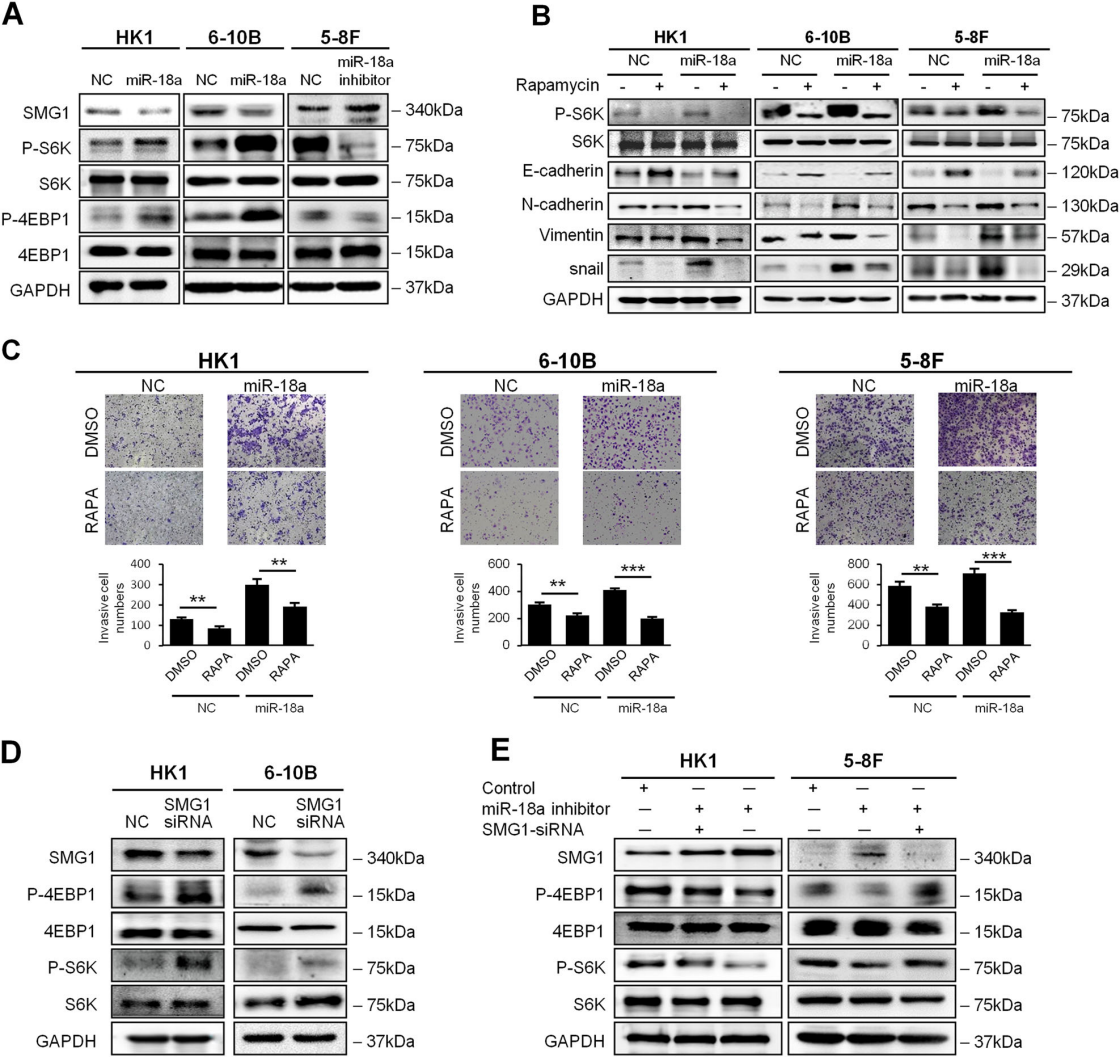
miR-18a exerts an oncogenic effect through the SMG1/mTOR pathway. **a** Western blotting revealed that miR-18a overexpression increased the expression level of phosphorylated S6K and 4EBP1 in HK1 and 6-10B cells, whereas miR-18a inhibitor treatment decreased the protein levels of p70S6k and p4EBP1 in 5-8F cells. **b** Inhibition of mTOR by rapamycin abolished the effects of miR-18a on EMT in HK1, 6-10B, and 5-8F cells. **c** The invasive ability of HK1, 6-10B, and 5-8F cells expressing miR-18a or the empty vector was evaluated by a Transwell assay with or without treatment with 20 ng/ml rapamycin. **d** Suppression of SMG1 expression by siRNA increased the expression levels of phosphorylated S6K and 4EBP1 in HK1 and 6-10B cells. **e** Suppression of SMG1 expression by siRNA in miR-18a inhibitor-treated HK1 and 5-8F cells rescued phosphorylated S6K and 4EBP1 expression. Data are presented as the mean ± SD. All assays were done in triplicate, and the values represent the mean of three independent experiments. *P < 0.05; **P < 0.01; ***P < 0.001

To examine the role of miR-18a expression in mediating the levels of activated mTOR, we detected the phosphorylated forms of downstream effectors in the AKT/mTOR signaling pathway by western blotting. Overexpression of miR-18a in HK1 and 6-10B cells upregulated the levels of p70S6K and 4EBP1 but had no effect on the total levels of the proteins. In contrast, knockdown of miR-18a in 5-8F cells dramatically reduced phospho-S6K and p-4EBP1 levels (Fig. 4a). To confirm that mTOR pathway activation was critical in miR-18a-promoted invasion and metastasis, we examined the effect of the mTOR inhibitor rapamycin on the enhanced invasion stimulated by miR-18a. The western blot assay showed that the EMT phenotype induced by miR-18a was suppressed by rapamycin treatment (Fig. 4b). In addition, the increased invasion of miR-18a overexpressing HK1, 6-10B, and 5-8F cells was suppressed by rapamycin treatment (20 ng/ml) (Fig. 4c). These findings confirmed that miR-18a promotes cellular motility and EMT in NPC cells through mTOR signaling.

To confirm that the activation of the mTOR pathway by miR-18a is indeed SMG1-dependent in NPC cells, we first examined the expression of the mTOR signaling-related molecules in 6-10B cells after RNAi-mediated silencing of SMG1. As expected, we observed that phosphorylated mTOR, p70S6K and 4EBP1 were significantly increased by SMG1 inhibition in NPC cells (Fig. 4d). Moreover, western blot analysis demonstrated the restoration of phosphorylated p70S6K and 4EBP1 by the inhibition of SMG1 in HK1 and 5-8F cells transfected with the miR-18a inhibitor (Fig. 4e). All of these findings confirmed that the oncogenic role of miR-18a in NPC cells depends on SMG1/mTOR signaling.

### Suppression of NPC cell growth by antagomir-18a in mouse xenografts

**Fig. 5:**
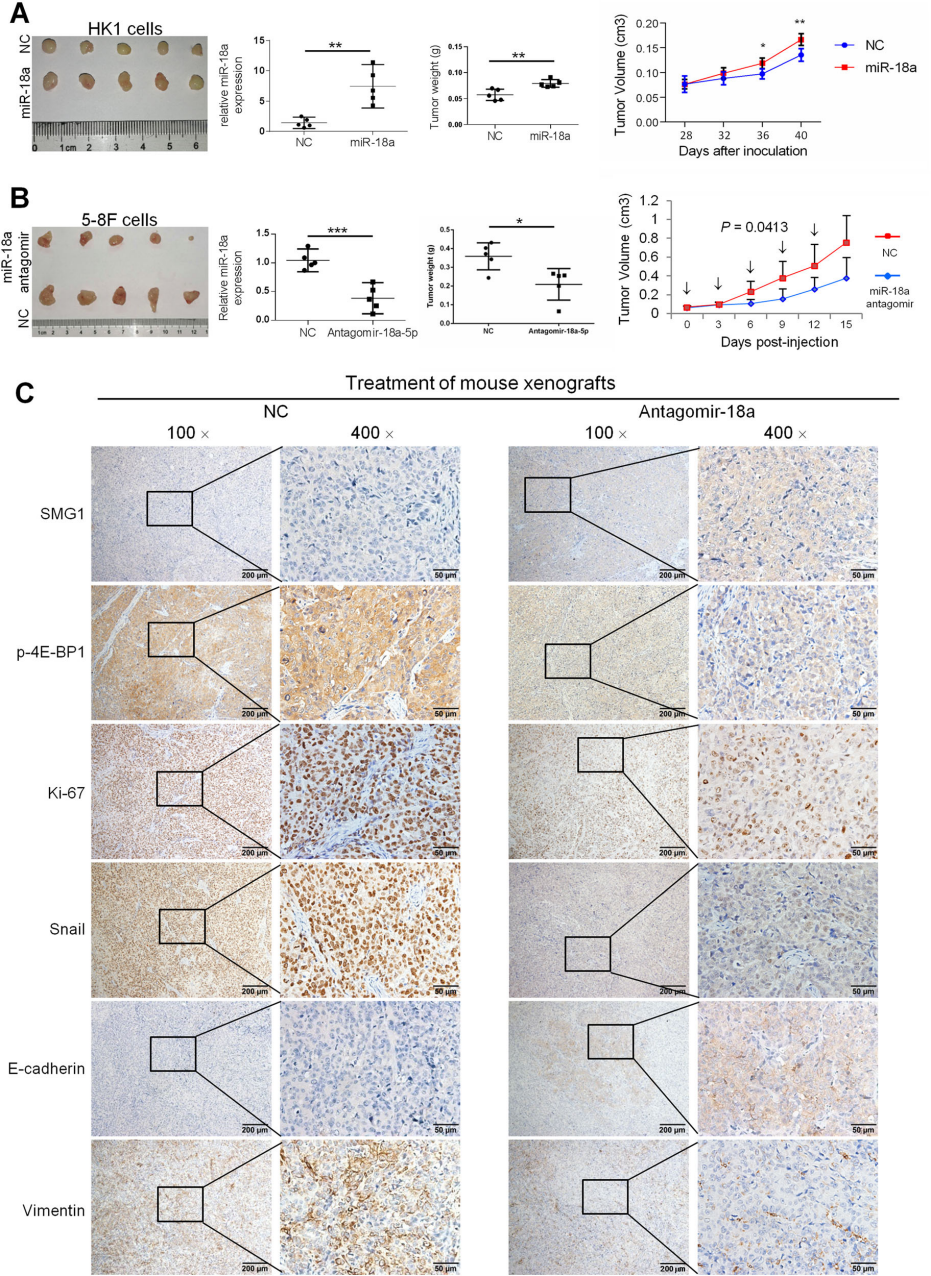
In vivo tumorigenesis was accelerated by miR-18a expression and inhibited by antagomir-18a-5p treatment. **a** miR-18a overexpression enhanced tumor growth in vivo. The left panel shows images of HK1/NC and HK1/miR-18a xenografts. The middle panels show quantification of miR-18a expression in xenografts generated from HK1-miR-18a or HK1-control cells by using qRT-PCR assay and the tumor weight at the completion of the experiment. Right, tumor growth curve. **b** Subcutaneous tumors formed from the injection of 5-8F cells into nude mice were treated with antagomir-18a-5p or control antagomir. Left, images of xenograft tumors generated from 5-8F cells at the end of the study in nude mice that received injections of antagomir-18a-5p or control antagomir (*n* = 5 per group). The middle panels show quantification of miR-18a expression in xenografts injected with antagomir-18a-5p or control antagomir by using qRT-PCR assay and the tumor weight at the completion of the experiment. Right, tumor growth curve. Injections of the antagomirs are indicated by arrows. Repeated measure analysis of variance with Greenhouse–Geisser correction applied (due to rejection of Mauchly’s Test of Sphericity) was used to compare the tumor volume in the two groups during the six subsequent measurements. bars, SD. ***P < 0.001. **c** Representative images showing a marked increase in SMG1 and E-cadherin protein levels and a decrease in Snail, p-4EBP1, Vimentin and Ki-67 levels in the antagomir-18a-5p-treated group compared with the control group of xenograft tumors. Magnification: ×100 and ×400

To further confirm the growth-stimulating effect of miR-18a, we conducted an in vivo tumor formation experiment by subcutaneously injecting HK1-miR-18a or the control cells into nude mice. At 40 days after implantation, the mice injected with HK1-miR-18a cells had significantly heavier tumor burdens than their controls (Fig. 5a).

The above results suggest that miR-18a might be a potential therapeutic target in NPC. To address this hypothesis, subcutaneous tumors formed by 5-8F cells in nude mice were treated with antagomir-18a injections once every 3 days for 2 weeks. The treatments, which started at 15 days post-tumor cell implantation, began to inhibit tumor growth after the second injection (Fig. 5b), with no obvious difference in body weight between the two groups of mice (data not shown). IHC staining on tumor sections showed a marked increase in SMG1 and E-cadherin protein levels and a decrease in Snail, p-4EBP1, Vimentin and Ki-67 protein levels in the antagomir-18a treated group (Fig. 5c). Interestingly, weaker staining of SMG1 was observed in the edges of the tumor masses than in the centers, in contrast with the staining of Ki-67, Snail and Vimentin, which was stronger in the tumor edges than in the centers (Supplementary Fig. 2).

### miR-18a can be induced by NF-κB activation and LMP1 expression in NPC cells

**Fig. 6:**
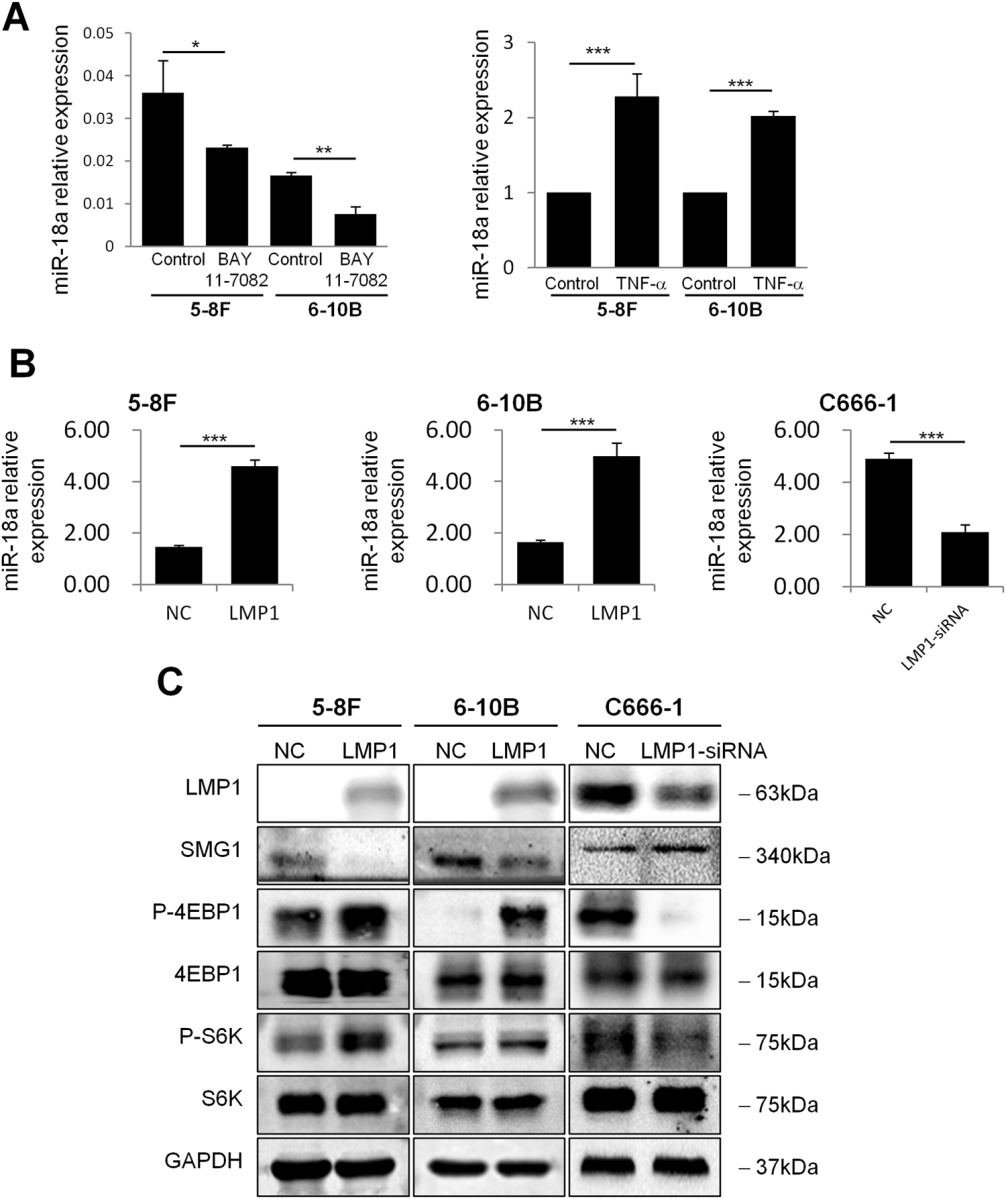
NF-κB activation and LMP1 expression induced miR-18a in NPC cells. 5-8F and 6-10B cells were treated with BAY 11-7082 (2.5 μM) (**a**) or TNF-α (10 ng/ml) (**b**) as indicated and then subjected to qRT-PCR analysis to determine miR-18a levels. **c** qRT-PCR analysis of miR-18a expression after transfection of LMP1-expressing plasmid in 5-8 F and 6-10B cells, or LMP1-specific siRNA in C666-1 cells. **d** Effects of LMP1 on SMG1 expression and mTOR activation were confirmed in 5-8F-LMP1, 6-10B-LMP1 and LMP1-kncok down C666-1 cells by western blot assays. Data are presented as the mean ± SD. All assays were done in triplicate, and the values represent the mean of three independent experiments. *P < 0.05; **P < 0.01; ***P < 0.001

Since multiple putative NF-κB binding sites were predicted in the promoter region of miR-18a, we suggest that NF-κB activation might regulate miR-18a expression. To identify the effect of NF-κB activity on miR-18a expression, BAY 11-7082, an NF-κB inhibitor, was used to inhibit IKKα/β activation, which leads to the translocation of p65 into the nucleus. The miR-18a expression levels were examined in NPC cells following pretreatment with BAY 11-7082 by qRT-PCR. As shown in Fig. 6a, decreased expression of miR-18a was observed in the BAY 11-7082-pretreated 5-8F and 6-10B cells. In contrast, the activation of NF-κB signaling by TNF-α treatment increased miR-18a levels in NPC cells (Fig. 6b). Moreover, dual-luciferase reporter assay demonstrate that TNF-α induced NF-κB activation increased luciferase activity in NPC cells transfected with the luciferase constructs that encompass the putative NF-κB binding site at −827 or −1442 compared with the PGL4-basic construct. Luciferase activity of the construct containing the binding site -1698 was also elevated but did not reach statistical significance (*P* = 0.0793) (Supplementary Fig. 3). On the contrary, NF-κB inhibitor BAY 11-7082 treatment repressed luciferase activity of the constructs spanning the putative NF-κB binding site at −827, −1442 or −1698. Our results suggest that NF-κB activation might induce miR-18a expression by binding to the promoter regions of miR-18a.

It was known that LMP1 engages signaling proteins in the tumor necrosis factor receptor family and leads to the activation of NF-κB. Therefore, we set out to examine whether LMP1 affects the expression of miR-18a. The EBV-negative NPC cell lines 5-8Fand 6-10B were transiently transfected with the pcDNA3.1-LMP1 plasmid (a kind gift from Dr. Bi-Jun Huang from Cancer Center, SYSU) or an empty plasmid, and C666-1, an EBV-positive NPC cell line, was transfected with LMP1-specific siRNA. Quantitative RT-PCR analysis demonstrated elevated expression of miR-18a in LMP1-transfected cells compared to control cells, whereas suppression of miR-18a was detected in siLMP1-transduced C666-1 cells compared to control cells (Fig. 6c); these results suggest that LMP1 might induce the expression of miR-18a in NPC cells.

To further confirm the effects of LMP1 expression on SMG1 expression and mTOR activation in NPC cells, LMP1 was expressed in 5-8F and 6-10B cells by transfection of pcDNA3.1-LMP1 plasmid and inhibited in C666-1 cells by siRNA-LMP1 transfection, respectively. Western blot assays showed that phosphorylated p70S6K and 4EBP1 were increased after LMP1 expression and decreased after LMP1 inhibition (Fig. 6d).

## Discussion

The role of miR-18a in tumor progression remains controversial. miR-18a has been proposed to promote cancer progression in colon cancer and NPC^12,15^ but to inhibit tumor growth in glioblastoma, colorectal cancer, and basal-like breast cancer^16–18^. Although miRNA microarray assays have demonstrated the upregulation of miR-18a in NPC samples, the exact function of miR-18a in NPC cells has not yet been fully defined. A recent study reported that NPC cells transfected with miR-18a exhibited significantly decreased expression of DICER1 mRNA and protein but significantly increased proliferative and invasive properties compared to control cells, and the authors suggested that miR-18a expression might promote the proliferation and metastasis of NPC cells by regulating DICER1^12^. The downregulation of DICER1 mRNA by 2.56-fold in miR-18a-overexpressing 6-10B cells compared with control cells was also confirmed by the microarray assay in the present study (data not shown). However, which oncogenic pathways were regulated by miR-18a and mediated the oncogenic functions of miR-18a in NPC was still unclear.

Here, we demonstrate that enforced expression of miR-18apromoted NPC cell proliferation, invasion and EMT, whereas miR-18a knockdown produced the opposite effects. Moreover, qRT-PCR analysis demonstrated that miR-18a was significantly overexpressed in NPC patients and was associated with tumor size and TNM stage, a finding that is in line with those of previous microarray studies showing that miR-18a is overexpressed in NPC tissues and correlates with poor prognosis^9–11^.

To identify the genes correlated with miR-18a in NPC, we analyzed genes differentially expressed after miR-18a overexpression or knockdown by whole-genome microarray analysis. KEGG pathway analysis revealed top-scoring enrichment of the PI3K/AKT/mTOR pathway. One of the most important findings here is that SMG1, which is a recently characterized member of the phosphoinositide 3-kinase-related kinases family and has been previously identified to be a critical component of nonsense-mediated mRNA decay (NMD), was verified as a functional target of miR-18a. Despite the involvement of SMG1 in NMD, the role of SMG1 in carcinogenesis has been explored in recent years. Haploinsufficiency for SMG1 predisposes mice to the formation of a range of tumors, suggesting that SMG1 might be a key tumor suppressor^19^. Additionally, decreased SMG1 expression has been shown in acute myeloid leukemia (AML), colorectal cancer, hepatocellular carcinoma and HPV-positive head and neck squamous cell carcinoma cells^20–24^. However, to date, the expression and function of SMG1 in NPC have not been reported. Here, we demonstrated for the first time that SMG1 expression is inhibited by miR-18a and is significantly reduced in NPC tissues. Importantly, we identified SMG1 as a potential negative regulator of EMT and cell invasion in NPC, suggesting a tumor-suppressive role of SMG1 in NPC. Moreover, the immunohistochemical analyses in our mice xenograft tumor models showed the intense Ki-67, Snail and Vimentin staining at the edge of tumors, a region where tumor cells were considered to have higher growth and invasive potential. By contrast, a decreased number of SMG1-expressing cells was observed at the edges compared with the central portions of the tumors, supporting the suppressive role for SMG1 in advancing tumor growth in NPC.

Consistent with our microarray data, immunoblotting analysis confirmed that the overexpression of miR-18a increased the phosphorylation of the mTOR downstream effectors p70S6K and 4EBP1, while the knockdown of endogenous miR-18a exerted opposite effects. Since mTOR pathway was commonly over-activated and promoted cell invasion and metastasis in NPC, rapamycin treatment was supposed to reduce EMT and invasion ability in both NC and miR-18a-expressing groups (as shown in Fig. 4b, c)^25^. Upon rapamycin treatment, the induced invasion ability of 5-8F and 6-10B cells by miR-18a was almost completely abrogated (Fig. 4c), indicating that the mTOR pathway mediates the oncogenic role of miR-18a in NPC. However, miR-18a induced invasion in HK1 cells was only partially reduced by rapamycin treatment comparing with NC (Fig. 4c), which indicates that miR-18a might enhance HK1 cell invasion through other pathway besides mTOR. Actually, it has been reported previously that miR-18a regulate EMT and metastasis by targeting SMAD2^26^, DICER1^12^, CDC42^17^ or HIF1A^18^. Thus, miR-18a might induce NPC cell invasion through multiple targets.

Both SMG1 and mTOR belong to the phosphoinositide 3-kinase-related kinases family, and an antagonistic interaction between SMG1 and mTOR signaling was suggested by recent studies. Cristina et al. first reported that SMG1 and mTORC1 act antagonistically in planarian flatworms^21^. Later, Du et al. verified a negative correlation between mTOR and SMG1 expression levels in AML patients^20^. In the present study, we confirmed that the phosphorylation of mTOR signaling pathway molecules was upregulated by SMG1 knockdown. Moreover, the suppression of the mTOR pathway by miR-18a inhibitor was abolished upon knockdown of SMG1, suggesting that miR-18a regulated the mTOR signaling pathway in an SMG1-dependent manner. The above findings confirmed that miR-18a exerts its oncogenic roles through SMG1 inhibition and mTOR activation in NPC.

In this study, NF-κB activation was suggested to induce miR-18a. The expression of miR-18a was increased after TNF-α treatment and decreased in NF-κB inhibitor BAY 11-7082 treated NPC cells. Indeed, direct binding of the NF-κB p65 subunit to the promoter element of the miR-17-92 gene has been confirmed by chromatin immunoprecipitation and luciferase reporter assays in a previous study^13^. And in the present study, we verified by a dual-luciferase reporter gene system that NF-κB activation could induce miR-18a expression by binding to multiple putative binding sites in its promoter regions (Supplementary Fig. 3). NF-κB overexpression can be observed in almost all NPC tumors and plays an important role in NPC tumorigenesis^27^. As a prosurvival transcription factor, NF-κB has been reported to regulate sets of miRNAs in lung cancer, esophageal cancer and gastric cancer^28–30^. Interestingly, miR-18a has been reported to target the TNF-α-induced protein 3 (TNFAIP-3), an NF-κB pathway inhibitor and, thus, to be involved in the activation of NF-κB signaling^31^. Therefore, an NF-κB/miR-18a/NF-κB positive feedback loop might exist in NPC cells. Moreover, we showed that miR-18a expression was increased in LMP1-transfected NPC cell lines, and decreased in LMP1-knockdown C666-1 cells, suggesting that LMP1 might increase miR-18a expression in NPC cells. LMP1 mimics a constitutively activated tumor necrosis factor receptor, and induces transformation and promotes tumor progression by activating the NF-κB transcription factor pathway, the MAP kinase cascade, and the PI3K/AKT pathway in NPC cells^32–34^. Recent studies have shown that LMP1 regulates the aberrant expression of certain cellular miRNAs involved in tumorigenesis, including miR-146a and miR-155 in EBV-related B-cell malignancies^35,36^, miR-200 in EBV associated gastric carcinoma^37^ and miR-203 in NPC^38^. Our findings suggested that LMP1 might induce miR-18a expression through the NF-κB pathway. Since LMP1 has been reported to activate mTOR pathway in NPC cells, which is confirmed in this study (Fig. 6c), it raises a possibility that LMP1-induced mTOR activation might be mediated by miR-18a. However, the previous study showed that LMP1 activated mTOR signaling pathway through its upstream mediator PI3K/AKT in NPC^39^. Therefore, multiple signaling pathways might be involved in the regulation of mTOR by LMP1. Further experiments were needed to clarify the significance of miR-18a in the role of LMP1.

As shown in Table 1, no significant relation was found between miR-18a expression and EBV serological biomarkers of the NPC patients, which is not consistent with our finding that LMP1 upregulates miR-18a. It might be explained as follows: Firstly, EBV latent products were consistently detected in NPC tumors even in serological negative patients since and EBV DNA was known to reside in all the tumor cells of NPC. Secondly, almost all NPC tumors exhibit aberrant NF-κB activation, which can be triggered by pro-inflammatory stimuli or genetic mutations of upstream components in addition to LMP1 expression^40^. Lastly, previous studies showed that miR-18a can also be regulated by c-Myc, Notch signaling and E2F Transcription Factors^41,42^. Therefore, miR-18a expression might be upregulated through pathways in addition to LMP1-induced NF-κB activation.

Our in vivo study demonstrated that ectopic miR-18a could effectively enhance tumor growth in the pretreated mice xenograft models. More importantly, the therapeutic mice xenograft model demonstrated that synthetic antagomir-18a, which can efficiently and stably knockdown miR-18a specifically in living cells, could strongly block the tumor growth after the second intratumoral injection without significant toxicity. miRNA targeting therapy has attracted more and more attention and successfully used in multiple human diseases. Although NPC is radiosensitive, the prognosis for NPC is still poor even with the use of neoadjuvant therapy. Therefore, the development of new therapeutic approaches is warranted. Our study indicates that antagomir-18a may ultimately be clinically useful in the treatment of NPC.

In conclusion, our results suggest a novel oncogenic miR-18a/SMG1/mTOR axis and provide a rationale for investigation of miR-18a as a novel therapeutic target in NPC.

## Supplementary information

Supplementary Figure Legends

Supplementary Table 1

Supplementary Table 2

Supplementary Figure 1

Supplementary Figure 2

Supplementary Figure 3
