# Supplementary material for: MicroRNA-18a promotes cancer progression through SMG1 suppression and mTOR pathway activation in nasopharyngeal carcinoma
Source: Cell Death Dis. 2019 Oct 28;10(11):819. doi: 10.1038/s41419-019-2060-9 (PMC6817863; doi:10.1038/s41419-019-2060-9)
Supplement: Supplementary file 1 — Supplementary Figure Legends [file 41419_2019_2060_MOESM1_ESM.docx]

**Supplementary Figure 1. Quantitative real-time RT-PCR was performed to confirm the differentially expressed genes related with PI3K/AKT/mTOR or EMT network identified by the microarray assay.** FGF9, IL7, PIK3R3, AKT2 and EGFR genes were increased in 6-10B-miR-18a cells compared with control, and decreased after miR-18a inhibitor transfection. By contrast, SMG1, PPP2R1B, DLC1 and ATXN1 genes were decreased in 6-10B-miR-18a cells compared with control, and increased after miR-18a knockdown. The qRT-PCR results were generally consistent with the microarray data.

**Supplementary Figure 2. Weaker staining of SMG1 was observed in the edges of the xenograft tumors.** Immunohistochemistry showed a tendency toward weaker staining of SMG1, but stronger staining of Snail, Vimentin and Ki-67, at the tumor edge compared to the inner fields of the tumor. Magnification: × 100 and × 400.

**Supplementary Figure 3. Luciferase reporter analysis confirmed that NF-κB induce miR-18a expression.** 5-8F cells were transfected with different luciferase constructs spanning the putative NF-κB binding sites at -827, -1442 or -1698 upstream of the transcription start site of miR-17–92. Then cells were treated with TNF-α or BAY 11-7082 respectively for 24 h followed by measurement of luciferase activity. Luciferase activity was presented as the ratio of the activity of the test construct with the control pGL4-basic.
