# Supplementary material for: MicroRNA-18a promotes cancer progression through SMG1 suppression and mTOR pathway activation in nasopharyngeal carcinoma
Source: Cell Death Dis. 2019 Oct 28;10(11):819. doi: 10.1038/s41419-019-2060-9 (PMC6817863; doi:10.1038/s41419-019-2060-9)
Supplement: Supplementary file 2 — Supplementary Table 1 [file 41419_2019_2060_MOESM2_ESM.docx]

**Supplementary Table 1**

Sequences of PCR primers used for qRT-PCR in this study.

| **Gene** | **Forward primers (5'-3')** | **Reverse primers (5'-3')** |
| --- | --- | --- |
| FGF9 | GAAAGACCACAGCCGATTTG | TTCATCCCGAGGTAGAGTCC |
| IL7 | TTGGACTTCCTCCCCTGATCC | TCGATGCTGACCATTAGAACAC |
| PIK3R3 | GAGTATGGACCGCGATGA | TTGGCTTAGGTGGCTTTG |
| AKT2 | ACCACAGTCATCGAGAGGACC | GGAGCCACACTTGTAGTCCA |
| EGFR | AGGCACGAGTAACAAGCTCAC | ATGAGGACATAACCAGCCACC |
| SMG1 | CTGGCAACCCAGAACTGATAG | TGTAGCCACCCTTTTCGTCAT |
| PPP2R1B | CTTGTGTCAGTATTGCCCAGT | TGCTGCTTGTCGAAGTGTAGG |
| DLC1 | CCACGGACCTCCCATCTTC | GCTGTGCATACTGGGGGAA |
| ATXN1 | TCGGTGGAGCTTGGTTTACAA | GGGAGGACCCAATGAACTGG |
