# Supplementary material for: MicroRNA-18a promotes cancer progression through SMG1 suppression and mTOR pathway activation in nasopharyngeal carcinoma
Source: Cell Death Dis. 2019 Oct 28;10(11):819. doi: 10.1038/s41419-019-2060-9 (PMC6817863; doi:10.1038/s41419-019-2060-9)
Supplement: Supplementary file 3 — Supplementary Table 2 [file 41419_2019_2060_MOESM3_ESM.docx]

**Supplementary Table 2**

SMG1 expressionin miR-18a overexpressing or knockdown cells by the microarray assay.

|  | **Raw Intensity** | |  | **Normalized Intensity** | |  |  |
| --- | --- | --- | --- | --- | --- | --- | --- |
| **Gene** | **6-10B/NC** | **6-10B/miR-18a** |  | **6-10B/NC** | **6-10B/miR-18a** | **Regulation** | **Fold Change** |
| SMG1 | 3119.30 | 1230.25 |  | 10.94 | 9.86 | down | 2.12 |
| SMG1 | 3251.15 | 1311.51 |  | 11.00 | 9.95 | down | 2.07 |
| SMG1 | 9070.37 | 3953.63 |  | 12.57 | 11.49 | down | 2.12 |
|  | **5-8F/NC** | **5-8F/miR-18a-KD** |  | **5-8F/NC** | **5-8F/miR-18a-KD** |  |  |
| SMG1 | 330.36 | 1899.87 |  | 9.51 | 10.79 | up | 2.42 |
| SMG1 | 243.63 | 1624.46 |  | 9.03 | 10.55 | up | 2.87 |
| SMG1 | 88.38 | 447.37 |  | 7.37 | 8.77 | up | 2.63 |
|  |  |  |  |  |  |  |  |
