# Supplementary figures and images for: MicroRNA-18a promotes cancer progression through SMG1 suppression and mTOR pathway activation in nasopharyngeal carcinoma
Source: Cell Death Dis. 2019 Oct 28;10(11):819. doi: 10.1038/s41419-019-2060-9 (PMC6817863; doi:10.1038/s41419-019-2060-9)

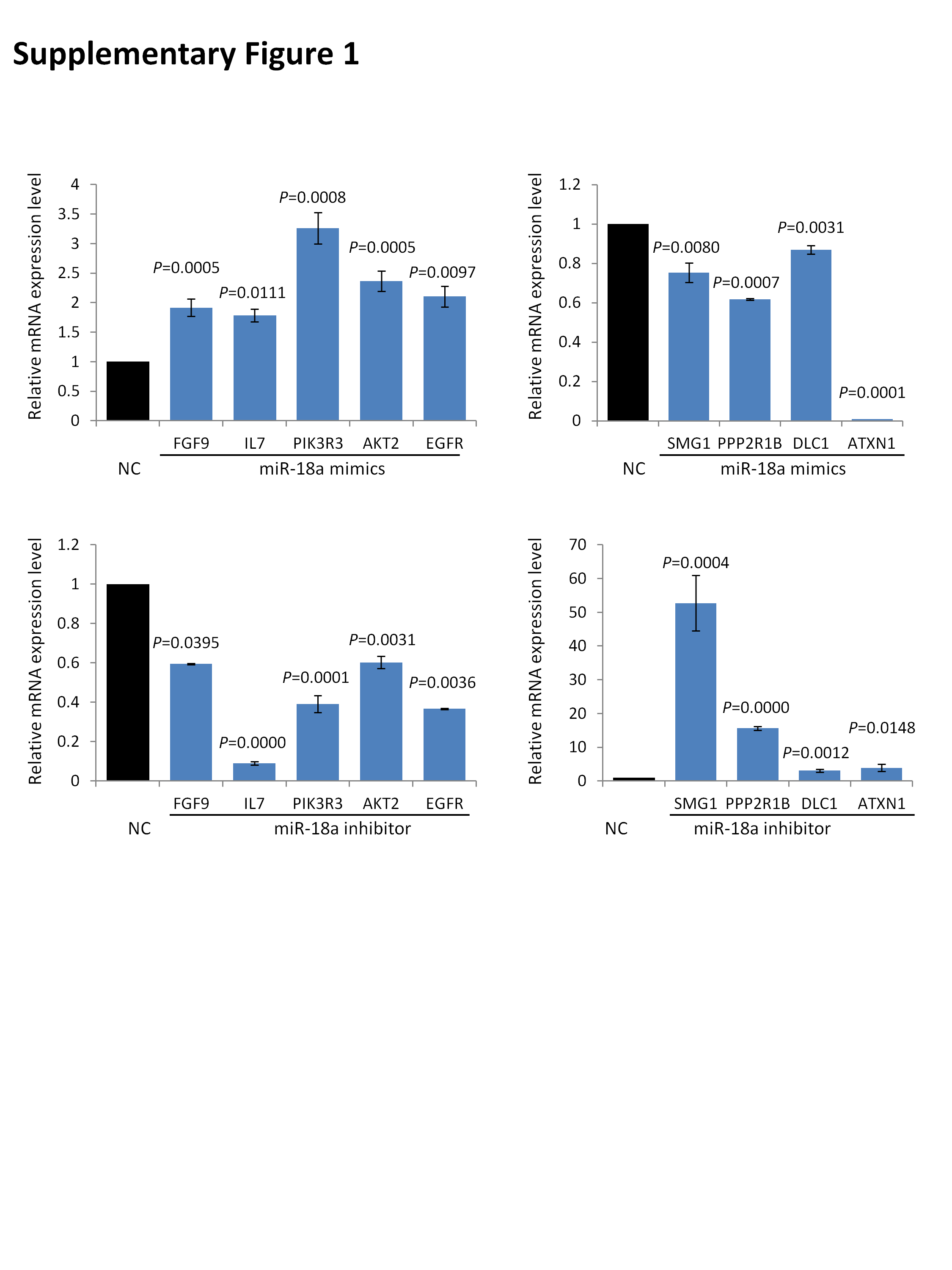

Supplement: Supplementary file 4 — Supplementary Figure 1 [file 41419_2019_2060_MOESM4_ESM.tif]

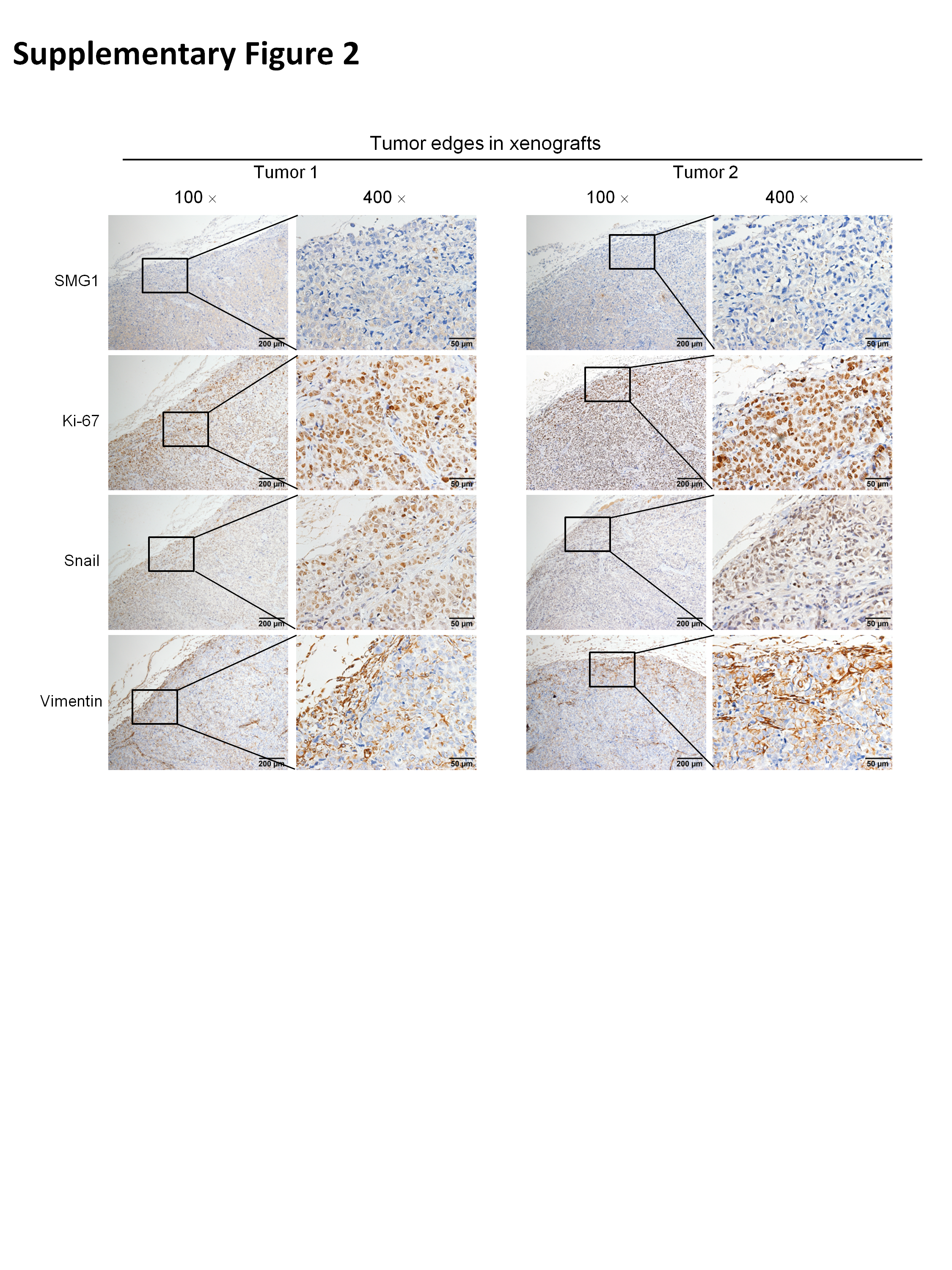

Supplement: Supplementary file 5 — Supplementary Figure 2 [file 41419_2019_2060_MOESM5_ESM.tif]

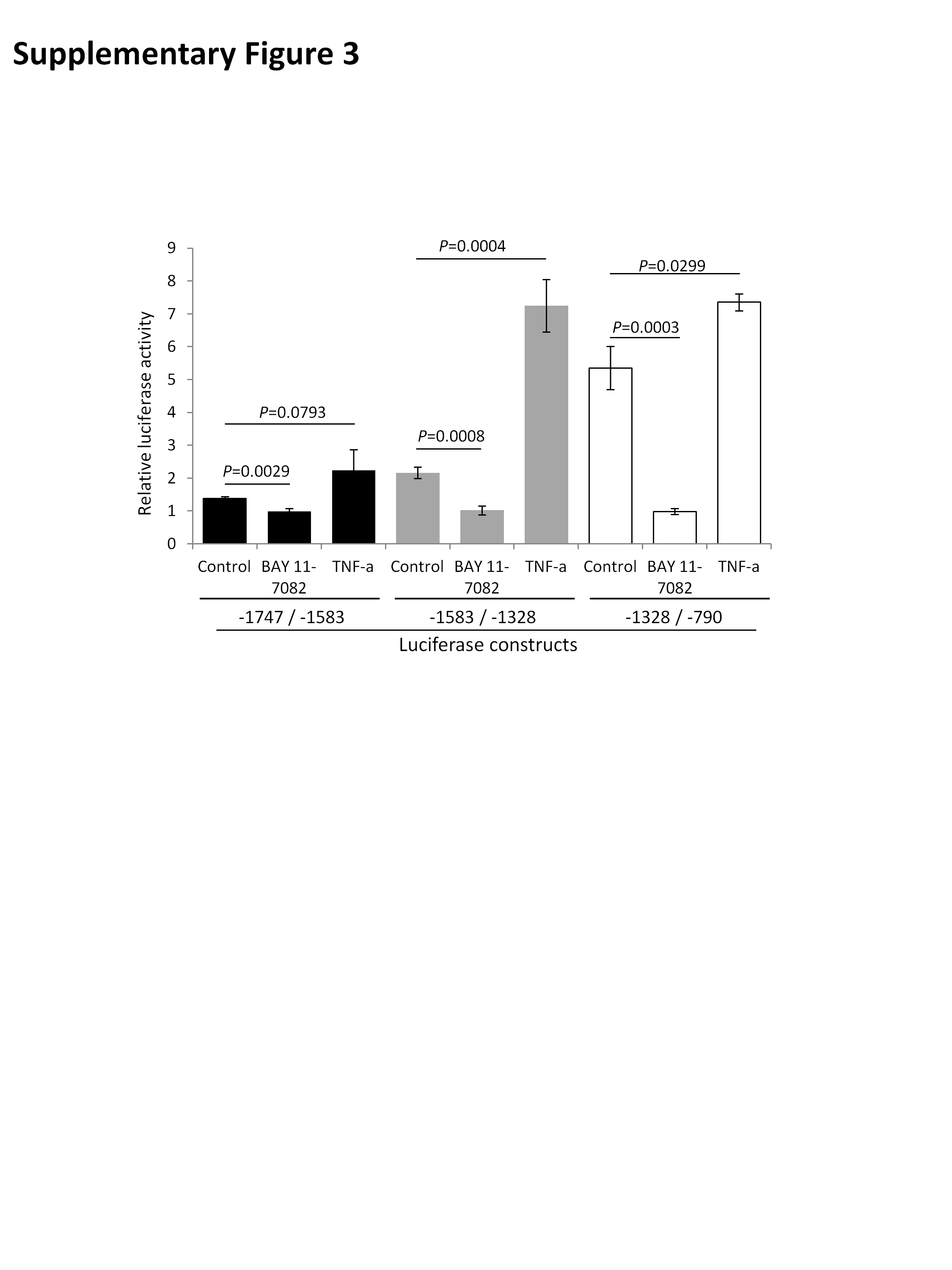

Supplement: Supplementary file 6 — Supplementary Figure 3 [file 41419_2019_2060_MOESM6_ESM.tif]
